# Abdominal Ultrasound and Abdominal Radiograph to Diagnose Necrotizing Enterocolitis in Extremely Preterm Infants

**Published:** 2019-02-26

**Authors:** Talkad S. Raghuveer, Richa Lakhotia, Barry T. Bloom, Debbi A. Desilet-Dobbs, Adam M. Zarchan

**Affiliations:** 1 University of Kansas School of Medicine-Wichita, Department of Pediatrics, Division of Neonatology; 2 Wesley Medical Center, Wichita, KS; 3 University of Kansas School of Medicine-Wichita, Department of Diagnostic Radiology

**Keywords:** necrotizing enterocolitis, extremely preterm infants, diagnostic imaging, pneumatosis intestinalis, pneumoperitoneum

## INTRODUCTION

Necrotizing enterocolitis (NEC) is an important contributor toward mortality in extremely premature infants and Very Low Birth Weight (VLBW) infants. The incidence of NEC was 9% in VLBW infants (birth weight 401 to 1,500 grams) in the Vermont Oxford Network (VON, 2006 to 2010, n = 188,703).^1^ The incidence of NEC was 7% in 1993, increased to 13% in 2008, and decreased to 9% in extremely preterm infants (22 to 28 weeks gestation) in the Neonatal Research Network Centers (1993 to 2012).^2^ The incidence of surgically treated NEC varies from 28 to 50% in all infants who develop NEC.^3^ Surgical NEC occurred in 52% in the VON cohort.^1^ In this cohort, the odds of surgery decreased by 5% for each 100 gram increase in birth.

The incidence of surgical NEC has not decreased in the past decade.^4^ The mortality from NEC is significantly higher in infants who need surgery compared to those who did not (35% versus 21%).^1^ The case fatality rate among patients with NEC is higher in those surgically treated (23 to 36%) compared to those medically treated (5 to 24%).^3^ In addition to surgery, NEC mortality rates are influenced by gestational age, birth weight,^1^,^2^,^5^ assisted ventilation on the day of diagnosis of NEC, treatment with vasopressors at diagnosis of NEC, and black race.^6^,^7^

Extremely preterm infants who survive NEC are at risk for severe neurodevelopmental disability and those with surgical NEC have a significantly higher risk of such delays (38% surgical NEC versus 24% medical NEC).^8^ Diagnosis of necrotizing enterocolitis is challenging and it is usually suspected based on non-specific clinical signs. Bell’s criteria and Vermont-Oxford Network criteria help in the diagnosis of NEC.

Bell’s criteria, commonly used for diagnosis, staging, and planning treatment of NEC, were described in 1978 and modified in 1986.^9^,^10^ Bell’s stage I signs are non-specific: temperature instability, lethargy, decreased perfusion, emesis or regurgitation of food, abdominal distension, recurrent apnea, and on occasion, increased support with mechanical ventilation. Abdominal distension and emesis are more common than bloody stools in very preterm infants compared to term infants.^7^ Abdominal radiographic findings are an integral part of Bell’s criteria. Identification of Bell’s stage I NEC (early NEC) with abdominal radiograph is challenging, as the features on abdominal radiograph (normal gas pattern or mild ileus) are non-specific. With progression of NEC to Bell Stage IIA, the symptoms (grossly bloody stools, prominent abdominal distension, absent bowel sounds) and features on abdominal radiographs (one or more dilated loops and focal pneumatosis) are more specific.

On the other hand, the Vermont Oxford Network criteria for NEC consist of at least one physical finding (bilious gastric aspirate or emesis, abdominal distension or occult/gross blood in the stool in the absence of anal fissure) and at least one feature on abdominal radiograph (pneumatosis intestinalis, hepatobiliary gas, or pneumoperitoneum).^1^ These features correspond to Bell Stage IIA or Stage IIB and are not features of early NEC. Thus relying solely on abdominal radiograph for diagnosis of early NEC, as is practiced currently, has significant drawbacks especially in extremely premature infants.^7^ Ultrasound has been suggested to improve the percentage of infants diagnosed with early NEC.^11^ However, this imaging modality is not used routinely in the diagnosis or management of NEC.

As the incidence of surgical NEC and mortality from NEC continues to be high, the literature to demonstrate the shortcomings of abdominal radiographs and promise of abdominal ultrasound in diagnosis of NEC is reviewed.

## CHALLENGES OF USING ABDOMINAL RADIOGRAPHY TO DIAGNOSE NEC

### Negative abdominal radiographs

Despite developments in the field of imaging, abdominal radiograph continues to be standard of care to diagnose NEC. However, it has been known since the 1980s that premature infants developed significant gangrenous NEC (intestinal gangrene documented at surgery or autopsy) before abdominal radiograph was diagnostic of NEC.^12^ The sensitivity of abdominal radiographs was low to diagnose pneumatosis (44%), portal venous gas (13%), and free air (52%), but the specificity ranged from 92 to 100% in a cohort of preterm infants with NEC and some with spontaneous intestinal perforation confirmed at surgery.^13^ The authors concluded that negative radiologic findings need to be interpreted with caution in preterm infants suspected to have NEC or spontaneous intestinal perforation.

A higher percent of radiographs were negative for pneumatosis, portal venous gas, pneumoperitoneum in extremely premature infants (birth weight <1,000 grams) compared to term infants.^7^ Gasless abdomen as a feature of NEC was absent in term infants but occurred in 42% of extremely premature (EP) infants.

### Variability in interpretation of abdominal radiographs in preterm infants with NEC

Significant variation in the interpretation of abdominal radiographs to diagnose NEC, in preterm infants, has been reported.^14^ Two studies regarding inter-observer variability in the interpretation of abdominal radiographs in suspected NEC concluded that the management of infants with suspected NEC should not be based solely on radiologic signs and that abdominal radiographs were not sufficient to make a correct and timely diagnosis.^15^,^16^

More recently, Coursey et al.^17^ devised a 10-point scale of abnormal radiographic findings (Duke Abdominal Assessment Scale) and concluded that the inter-observer agreement was superior to earlier studies, but variation in interpretation persisted.

### Radiation exposure from multiple abdominal radiographs for NEC in extremely premature infants

Premature infants are exposed to multiple radiographs during their hospital stay and especially those suspected or diagnosed to have NEC. In the study by Iyer et al.,^18^ NEC was a significant risk factor associated with radiation exposure.

In summary, abdominal radiographs are not very sensitive to diagnose early NEC with a well-documented problem of inter-observer variability in interpretation. Extremely preterm infants are exposed to increasing doses of radiation from the multiple radiographs done to diagnose and monitor NEC. In addition, there is little evidence regarding the optimal frequency, whether to use one or two views, and how long to continue abdominal radiographs for diagnosis and monitoring progression of NEC.^19^ On the other hand, abdominal radiographs can be obtained any time; neonatologists and radiologists are familiar with the features of NEC on abdominal radiograph and a large number of extremely preterm infants have been diagnosed with NEC using abdominal radiographs for decades. Hence, abdominal radiographs continue to be the “gold standard” for diagnosis of NEC at this time.

## CHALLENGES OF USING ABDOMINAL ULTRASOUND TO DIAGNOSE NEC

Abdominal ultrasound provides real time images of bowel and fluid in the peritoneal cavity and can depict some features of NEC that cannot be seen with abdominal radiograph such as peristalsis and presence or absence of bowel wall perfusion.^11^ Abdominal ultrasound is possibly more sensitive, compared to abdominal radiograph, to detect intramural gas (pneumatosis), portal venous gas, and free gas (pneumoperitoneum).

### The technical challenges of using abdominal ultrasound (with Doppler and gray scale) to detect NEC

Efforts to evaluate the role of abdominal ultrasound for diagnosis of NEC date back to the 1980s.^20^–^22^ Recently, Faingold et al.^23^ showed that Doppler ultrasound could be used to study bowel viability in infants with NEC. Kim et al.^24^ showed the utility of using gray-scale ultrasound to evaluate the bowel wall in infants with NEC.

Three recent reviews detailed the usefulness of abdominal ultrasound in the diagnosis of NEC.^11^,^25^,^26^ Faingold et al.^23^ and Yikilmaz et al.^27^ give technical details regarding a thorough ultrasound examination of the abdomen in suspected cases of NEC in preterm infants. These details would help other physicians to develop protocols for use of abdominal ultrasound to diagnose NEC. The initial learning curve regarding the techniques of abdominal ultrasound to detect NEC and the interpretation of images may be steep.

Intramural gas (pneumotosis) consisting of gas bubbles in the sub-serosal and submucosal layers of the bowel wall appear as echogenic dots.^25^ The foci of dots from pneumatosis may vary from a few dots to multiple dots involving the whole circumference of bowel.

Portal venous gas is seen as echogenic round particles in the parenchyma of the liver (trapped in small branches of portal vein).^25^ Free air (pneumoperitoneum) is seen as bright echoes between the abdominal wall and the anterior surface of the liver. Abdominal fluid usually is detected close to the bladder and localized echogenic fluid is suggestive of bowel perforation.

### Usefulness of abdominal ultrasound in diagnosing NEC in preterm infants

In the Kim et al.^24^ study, detection of pneumatosis using abdominal ultrasound was more accurate than abdominal radiographs in early NEC (Bell stage I). In a prospective study, Dordelmann et al.^28^ screened premature infants (796 were routine ultrasound screening without abdominal symptoms and 48 screening for suspected NEC) for portal venous gas using abdominal ultrasound. None of the 796 routine screenings were positive. The study concluded that no other clinical condition other than NEC was associated with visualization of portal venous gas on abdominal ultrasound.

In a retrospective study from the same group of investigators, the use of abdominal ultrasound to detect portal venous to diagnose NEC showed a sensitivity of 82% and specificity of 96%.^29^ One infant with volvulus was diagnosed with portal venous gas and was deemed a false positive.

Detection of portal venous gas on abdominal radiographs has been a harbinger of bad outcomes.^30^ It is possible that abdominal ultrasound may identify portal venous gas before extensive necrosis of the intestines has occurred.^25^ Inadvertent injection of air either during the placement or use of an umbilical venous catheter has been reported as a benign cause of portal venous gas,^31^,^32^ but most often portal venous gas is diagnostic of NEC.

In a recent meta-analysis of abdominal ultrasound studies in infants with suspected NEC (11 studies, n = 748 infants), Cuna et al.^19^ have shown that the bowel ultrasound features strongly associated with surgery or death were free air, absent peristalsis, complex ascites, and focal fluid collections. They also found that some bowel ultrasound features were not associated with surgery or death such as portal venous gas, increased bowel perfusion, and simple ascites. Chen et al.^33^ found that abdominal ultrasound was significantly superior to abdominal radiograph in the prognostic prediction of NEC.

### Limitations of abdominal ultrasound for diagnosis of NEC in preterm infants

The use of abdominal ultrasound for diagnosis of NEC has some technical limitations, especially to assess perfusion of the bowel, and some of these limitations may be overcome in the future with the use of contrast-enhanced ultrasound.^34^

The experience of the radiologist and the sonographer to assess and interpret the abdominal ultrasound for features of NEC is important. In addition, there were no studies to assess the inter-observer and intra-observer variability of interpretation of abdominal ultrasound findings in NEC.^25^

Abdominal radiographs are available round the clock in most neonatal intensive care units. However, abdominal ultrasound may not be available outside of regular hours in most hospitals. Interpretation of abdominal radiographs to diagnose NEC is part of the training for radiology residents and neonatal fellows. It is not clear how many academic programs provide training in interpretation of abdominal ultrasound for diagnosis of NEC to radiology residents and neonatal fellows.

In summary, there is evidence that abdominal ultrasound is useful in diagnosing NEC with a number of limitations as outlined above. There is no evidence for optimal timing and frequency of abdominal ultrasound imaging in suspected cases of NEC. Finally, it is not clear from studies done so far whether the combined imaging (abdominal radiograph plus abdominal ultrasound) would improve outcomes (surgery and/or death) in extremely preterm infants suspected to have NEC.

All studies so far have compared abdominal radiograph with abdominal ultrasound to diagnose NEC. However, a well-designed, randomized, prospective, multi-center clinical trial comparing abdominal ultrasound plus abdominal radiographs to abdominal radiographs alone in extremely preterm infants suspected to have NEC has not been done. This type of study would be important as other efforts toward early identification of NEC in extremely preterm infants, including biomarkers and near-infrared spectroscopy (NIRS), have not yielded clinically significant results.^4^

## CONCLUSIONS

Surgical NEC occurs in a high percent of extremely preterm infants with NEC leading to increased mortality and long-term morbidity in these infants.^1^,^3^ Primary prevention of NEC in extremely preterm infants is of utmost important and so are the efforts to decrease surgical NEC. Abdominal radiographs are the current standard for diagnosing NEC but have significant limitations in diagnosing early NEC.^7^ Abdominal ultrasound has shown promise in diagnosis and prognosis of NEC.^33^ There is an urgent need to decrease surgical NEC and mortality in extremely preterm infants with NEC and a well-designed study comparing abdominal radiograph alone versus simultaneous abdominal radiograph and abdominal ultrasound may help in this regard.
